# FRET analysis of HIV‐1 Gag and GagPol interactions

**DOI:** 10.1002/2211-5463.12328

**Published:** 2017-10-19

**Authors:** Shimon Takagi, Fumitaka Momose, Yuko Morikawa

**Affiliations:** ^1^ Kitasato Institute for Life Sciences and Graduate School for Infection Control Kitasato University Tokyo Japan; ^2^ Present address: A2 Healthcare Corporation Sumitomo Fudosan Korakuen Bldg., Koishikawa 1‐4‐1, Bunkyo‐ku Tokyo 112‐0002 Japan

**Keywords:** assembly, Förster/fluorescence resonance energy transfer, Gag, GagPol, HIV‐1

## Abstract

The Gag protein of HIV multimerizes to form viral particles. The GagPol protein encoding virus‐specific enzymes, such as protease, reverse transcriptase, and integrase, is incorporated into HIV particles via interactions with Gag. The catalytically active forms of these enzymes are dimeric or tetrameric. We employed Förster resonance energy transfer (FRET) assays to evaluate Gag–Gag, Gag–GagPol, and GagPol–GagPol interactions and investigated Gag and Pol interdomains tolerant to fluorescent protein insertion for FRET assays. Our data indicated that the matrix (MA)–capsid (CA) domain junction in the Gag region and the Gag C terminus were equally available for Gag–Gag and Gag–GagPol interaction assays. For GagPol dimerization assays, insertion at the MA–CA domain junction was most favorable.

**Abbreviations**
CA: capsid
CTD: C‐terminal domain
EGFP: enhanced green fluorescent protein
FRET: Förster/fluorescence resonance energy transfer
HIV‐1: human immunodeficiency virus type 1
IN: integrase
MA: matrix
MHR: major homology region
mSB: mStrawberry
NC: nucleocapsid
NTD: N‐terminal domain
PR: protease
RT: reverse transcriptase

The Gag protein of HIV‐1 is viral capsid (CA) precursor protein containing the N‐terminal matrix (MA), the central CA, the nucleocapsid (NC), and the C‐terminal p6 domains and drives viral particle assembly. The GagPol protein is synthesized by ribosomal frameshifting, a mechanism by which ribosomes slip backward in the *gag* gene and shift to the *pol* reading frame, resulting in MA, CA, and NC in the Gag region and the transframe domain p6*, protease (PR), reverse transcriptase (RT), and integrase (IN) in the Pol region.

Retroviral Gag has key roles in particle assembly. In many cell types, the expression of HIV‐1 Gag alone produces a Gag virus‐like particle, similar to immature HIV‐1 1. In contrast, the GagPol protein is incapable of binding to the membrane 2 and is only incorporated into an HIV‐1 virion by interactions, termed ‘coassembly’ with Gag 3. As GagPol is a precursor protein that is processed to produce the virus‐specific enzymes, PR, RT, and IN, the incorporation of GagPol into HIV‐1 particles is absolutely required for the virion infectivity.

Previous mutation studies have identified three discrete Gag regions responsible for particle assembly: MA, the CA C‐terminal domain (CTD) to NC, and p6. MA is a membrane‐binding domain and itself forms an MA trimer 4. The CA CTD to NC is a main assembly domain that promotes Gag multimerization. Structural studies have revealed that CA helix 9, located on the CA CTD, forms the CA dimer interface by parallel packing 5 and two amino acid substitutions on helix 9 (W184A and M185A) disrupt particle assembly 6. Structural studies have also revealed that CA forms a hexamer ring through the CA N‐terminal domain (NTD) and that the CA CTD stabilizes the CA assembly 7, 8, 9. NC is an HIV RNA‐binding domain and promotes Gag multimerization by serving as the RNA scaffold 10, 11. In fact, the RNA–NC interaction promotes a low level of Gag oligomerization by the CA CTD 11. p6 is termed the late domain and facilitates particle budding. All of these Gag regions individually contribute to virion assembly, but the CA CTD to NC is absolutely required for Gag assembly.

Gag–GagPol interactions have similarly been investigated by mutation studies in which mutant GagPol was incorporated into virus particles by coassembly with Gag. These studies have indicated that the CA major homology region (MHR) and the adjacent C‐terminal CA region in the CA CTD 12, 13, 14 within GagPol are responsible for coassembly with Gag. It is plausible that Gag and GagPol interact through CA helix 9, similar to Gag–Gag interactions, but this has not yet been proven.

The PR, RT, and IN are synthesized as a portion of the GagPol precursor. The PR embedded in the Pol region is activated upon GagPol dimerization and is autocatalytically cleaved to become a fully active PR dimer 15. As dimerization of PR is essential for PR activity, dimerization of the GagPol precursor is likely to be a prerequisite for this process. However, not only PR but also RT and IN form dimers and tetramers, respectively 16, 17, 18, suggesting that several independent dimerization interactions participate in the dimerization of the GagPol precursor. It is also possible that the Gag region in GagPol drives GagPol–GagPol interactions. Thus, the region responsible for the dimerization of GagPol precursors is unclear.

Förster fluorescence resonance energy transfer (FRET) assays are used in molecular virology. In HIV studies, Gag–Gag interactions 19, 20, 21, PR dimerization 22, 23, and RT heterodimerization 24 have been reported with various donor–acceptor FRET pairs. As energy transfer in FRET assays occurs when the donor and acceptor fluorescent proteins are very close and nearly attached to each other (< 5 nm), the assays are reliable for the detection of direct protein–protein interactions. We have previously established FRET assays with Gag/Pol‐enhanced green fluorescent protein (EGFP) and Gag/Pol‐mStrawberry (mSB), in which EGFP and mSB were placed at the PR–RT junction containing inactive PR. These constructs express GagPol with EGFP or mSB and authentic Gag and are available for the detection of GagPol dimerization 25, 26. In the present study, to measure not only homotypic (Gag–Gag and GagPol–GagPol) but also heterotypic (Gag–GagPol) interactions, we placed fluorescent proteins at various domain junctions within Gag and Pol and assessed the availability of the Gag‐ and GagPol‐fluorescent constructs for Gag–Gag, Gag–GagPol, and GagPol–GagPol interactions in FRET assays.

## Materials and methods

### DNA construction and transfection

All derivatives used in this study were from the HIV‐1 molecular clone pNL4‐3 and contained inactive PR (D25N mutation). The derivative expressing Gag with a FLAG peptide at the C terminus without GagPol and that expressing GagPol with a HA peptide at the C terminus in which the *gag* and *pol* genes were placed in‐frame by deleting the frameshifting signal (frameshift mutation) were described previously 2.

For FRET imaging, the pNL4‐3 derivatives expressing Gag(p6/F) (F shows EGFP or mSB) have been described previously 25, 26. The cDNAs encoding EGFP and mSB were inserted in‐frame at the MA–CA and p2–NC junctions in the pNL4‐3 derivative containing stop codons in the *pol* frame, leading to the expression of Gag(MA/F/CA) and Gag(p2/F/NC), without the *pol* gene products. For the expression of the GagPol precursor, cDNAs encoding EGFP and mSB were inserted in‐frame at the MA–CA, p2–NC, p6*–PR, and PR–RT junctions in the pNL4‐3 derivative containing the frameshift mutation and the D25N mutation in PR. The sequences corresponding to I153‐Q219 in the CA CTD and 5N‐R52 in NC were deleted from the pNL4‐3 derivatives expressing GagPol(MA/F/CA) (ΔCA CTD and ΔNC, respectively). For the disruption of CA dimerization, the mutations W184A and M185A 6 were introduced into the CA CTD. The stem loops 1 and 3 (SL1 and SL3) present in the 5′‐untranslated region, corresponding to the dimerization and packaging signals for the HIV genome 10, 27, were deleted (ΔSL1,3).

HeLa cells were transfected with the pNL4‐3 derivatives at a donor‐to‐acceptor ratio of 1 : 1 and were subjected to FRET analysis. In some experiments, HeLa cells and HIV‐1 particles in the culture supernatants were subjected to western blotting with an anti‐HIV‐1 p24CA monoclonal antibody.

### FRET analysis

Confocal images were acquired in three combinations (TCS‐SP5; Leica, Wetzlar, Germany): excitation/emission wavelengths of 488/500–560 nm (donor EGFP channel), 561/570–630 nm (acceptor mSB channel), and 488/570–630 nm (FRET channel). Thirty EGFP/mSB‐double‐positive cells from at least three independent experiments were subjected to FRET analyses. Three EGFP/mSB‐double‐positive regions per cell were randomly selected, and their FRET values were measured using FRET SE Wizard (Leica). The FRET efficiency was calculated according to the following previously described formula: *E*_A_ = (*B *− *A*β* *− *C* (γ* *− αβ))/(*C* (1* *− βδ)) 28. *A*,* B*, and *C* are the fluorescence intensities of the donor, FRET, and acceptor channels, respectively. The parameters α, β, γ, and δ are the calibration factors generated by donor‐only and acceptor‐only references and corrected for acceptor cross‐excitation cross talk, donor cross talk, acceptor cross‐excitation, and FRET cross talk, respectively. Intergroup comparisons were performed using unpaired *t*‐tests (parametric group analysis).

## Results

### FRET of Gag containing the fluorescent protein at the MA–CA and p2–NC junctions and the C terminus

**Figure 1:**
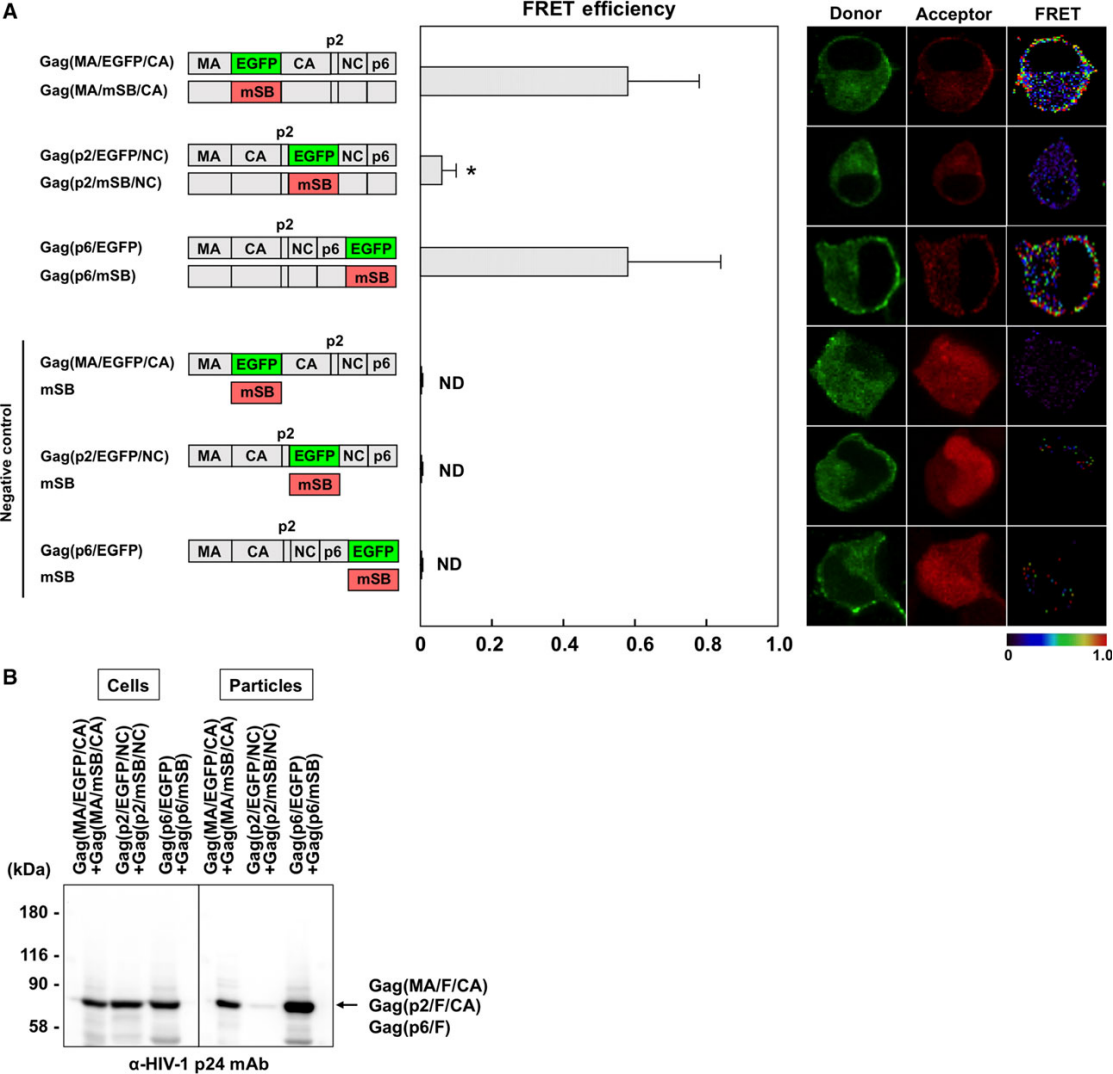
FRET assays for Gag–Gag interactions with Gag interdomain constructs and virus particle production. HeLa cells were cotransfected with a Gag(MA/F/CA), Gag(p2/F/NC), or Gag(p6/F) molecular clone pair. Combinations of Gag(MA/EGFP/CA), Gag(p2/EGFP/NC), or Gag(p6/EGFP) molecular clone and a plasmid expressing soluble mSB were used as negative controls. (A) FRET efficiencies of Gag interdomain constructs. The mean FRET efficiencies with standard deviations are shown. Their FRET efficiencies were statistically greater than those of the corresponding negative controls (*P *<* *0.01). *Statistically significant (*P *<* *0.01) compared with the Gag(MA/F/CA) pair. Representative FRET images are shown. FRET efficiencies are color‐coded with a color scale bar over a range of 0–1. (B) Virus particle production. The particle fractions purified from the culture supernatants of cells expressing the Gag interdomain constructs were analyzed by western blotting using a monoclonal antibody specific for HIV‐1 p24CA.

Several FRET studies have used Gag constructs fused to CFP and YFP at the C termini and showed that the majority of Gag–Gag interactions occurred at the plasma membrane 19, 21. We observed similar results using Gag constructs fused to EGFP/mSB at the C termini 26. The Gag domain junctions (e.g., MA–CA) are also available for the insertion of fluorescent proteins 20, 29. To compare the availability of these Gag constructs for FRET assays, we introduced EGFP and mSB to the MA–CA and p2–NC junctions (Fig. 1A). For comparison, pNL4‐3 derivatives expressing Gag fused to EGFP and mSB at the C terminus were used. Thus, we inserted EGFP and mSB at the same domain junctions in Gag, because Gag–Gag interactions occur by the homo‐oligomerization of each Gag domain and FRET requires the close proximity of donor and acceptor fluorescent proteins. When Gag(MA/EGFP/CA) and Gag(MA/mSB/CA) were coexpressed, the FRET signals were mainly detected at the plasma membrane (*E*_A_ = 0.58). An equivalent FRET efficiency was obtained using Gag(p6/EGFP) and Gag(p6/mSB) (*E*_A_ = 0.58). In contrast, when the fluorescent proteins were inserted into the p2–NC junction, the FRET efficiency was substantially lower (*E*_A_ = 0.06). Western blotting with virus particle fractions indicated that Gag(MA/F/CA) and Gag(p6/F), but not Gag(p2/F/NC), produced virus particles (Fig. 1B). These results were consistent with those of previous studies 19, 20, 21, 30, 31, 32.

**Figure 2:**
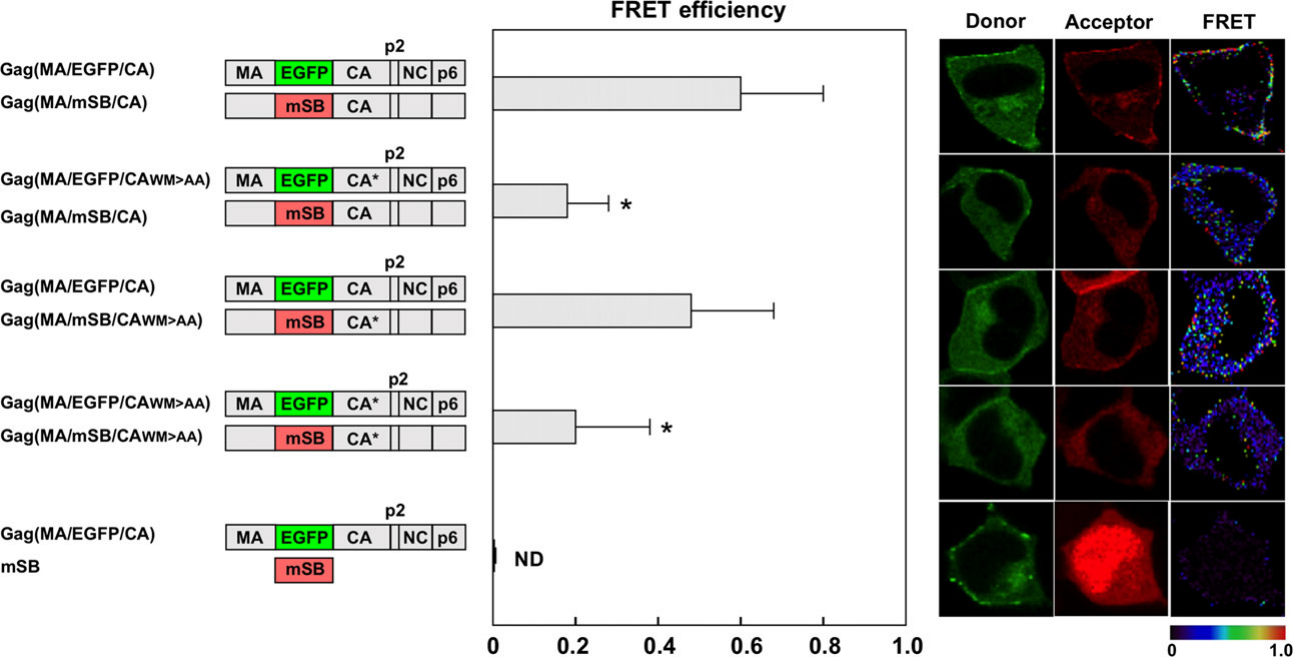
FRET assays with Gag(MA/F/CA) constructs containing CA dimer interface mutations. HeLa cells were cotransfected with a Gag(MA/F/CA) molecular clone pair containing CA mutations (W184A and M185A) in either donor or acceptor construct, or both. The FRET efficiencies were statistically greater than that of the negative control (Gag(MA/EGFP/CA) plus soluble mSB;* P *<* *0.01). *Statistically significant (*P *<* *0.01) compared with Gag(MA/F/CA). WM > AA, W184A and M185A. Representative FRET images are shown.

Previous studies have shown that the mutations W184A and M185A in the CA CTD dimer interface severely impair particle assembly 6. A FRET analysis with a Gag(p6/CFP) and Gag(p6/YFP) pair has revealed that these mutations abolish the Gag–Gag interaction 21. We examined these mutations with our Gag(MA/F/CA) construct (Fig. 2). When the mutations were introduced into both donor and acceptor Gag molecules, the FRET efficiency was reduced (*E*_A_ = 0.21). A similar level of reduction was observed when the mutations were present in the donor Gag alone (*E*_A_ = 0.19), but not in the acceptor Gag alone (*E*_A_ = 0.48). These results indicated that the insertion at the MA–CA junction was available for FRET analyses for Gag–Gag interactions, confirming a previous FRET study 20.

### FRET analysis of Gag–GagPol interactions

**Figure 3:**
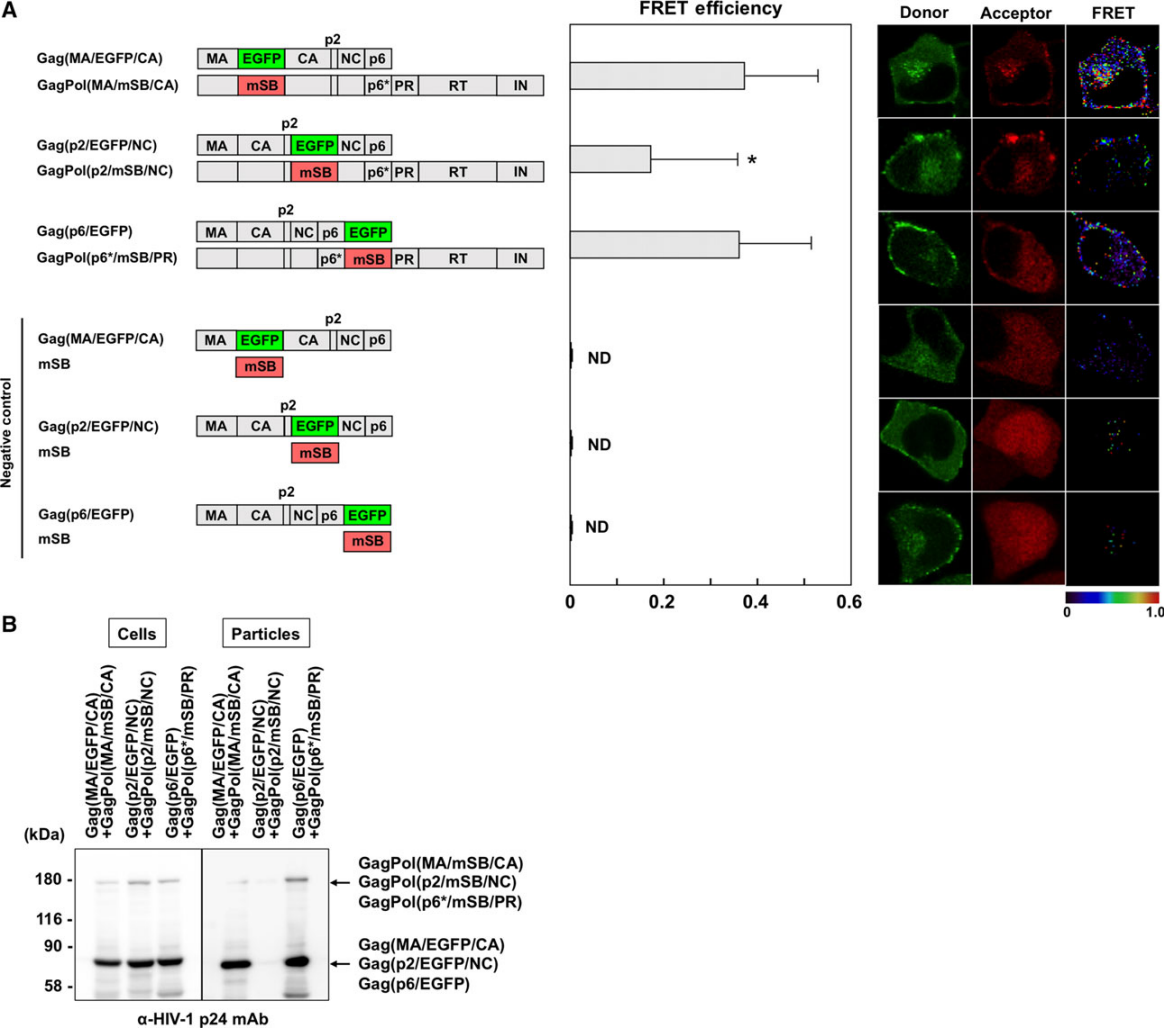
FRET assays for Gag–GagPol interactions with Gag interdomain constructs and virus particle production. (A) FRET efficiencies of Gag–GagPol with Gag interdomain fluorescent protein. HeLa cells were cotransfected with Gag(MA/EGFP/CA) plus GagPol(MA/mSB/CA), Gag(p2/EGFP/NC) plus GagPol(p2/mSB/NC), or Gag(p6/EGFP) plus GagPol(p6*/mSB/PR) molecular clone pair (a donor‐to‐acceptor DNA ratio of 1 : 1). Combinations of Gag(MA/EGFP/CA), Gag(p2/EGFP/NC), or Gag(p6/EGFP) molecular clone and a plasmid expressing soluble mSB were used as negative controls. The FRET efficiencies of the donor Gag–acceptor GagPol pairs were statistically greater than those of the corresponding negative controls (*P *<* *0.01). *Statistically significant (*P *<* *0.01) compared with the Gag(MA/EGFP/CA)–GagPol(MA/mSB/CA) pair. Representative FRET images are shown. (B) Virus particle production. HeLa cells were cotransfected with the Gag‐expressing and the GagPol‐expressing molecular clones at a DNA ratio of 10 : 1. The particle fractions purified from the cell culture supernatants were analyzed by western blotting using a monoclonal antibody specific for HIV‐1 p24CA.

We inserted EGFP into the domain junctions in Gag constructs (MA–CA and p2–NC junctions and C terminus) and mSB into the same Gag domain junctions within GagPol constructs (MA–CA, p2–NC, and p6*–PR junctions) containing the frameshift mutation and inactive PR (Fig. 3A). FRET requires equivalent levels of donor/acceptor expression, because a donor molecule transfers energy to an acceptor molecule in a one‐to‐one relationship. As we have previously shown that a 1 : 1 ratio of Gag to GagPol produced virus particles when the PR was inactive 25, the Gag‐ and GagPol‐fluorescent constructs were transfected to cells at a ratio of 1 : 1. The FRET efficiency for a combination of Gag(MA/EGFP/CA) plus GagPol(MA/mSB/CA) was ~ 0.37. The efficiency for Gag(p6/EGFP) plus GagPol(p6*/mSB/PR) was nearly equivalent (*E*_A_ = 0.36). In contrast, the coexpression of Gag(p2/EGFP/NC) and GagPol(p2/mSB/NC) showed a considerably lower FRET efficiency (*E*_A_ = 0.17), although it was still significantly different from that of the negative control. These results suggested that the MA–CA junction and the p6/p6* C termini were available for FRET analyses of Gag–GagPol interactions, similar to Gag–Gag interactions. Consistent with these results, western blotting indicated that GagPol(MA/mSB/CA) and GagPol(p6*/mSB/PR) were incorporated into virus particles (Fig. 3B).

**Figure 4:**
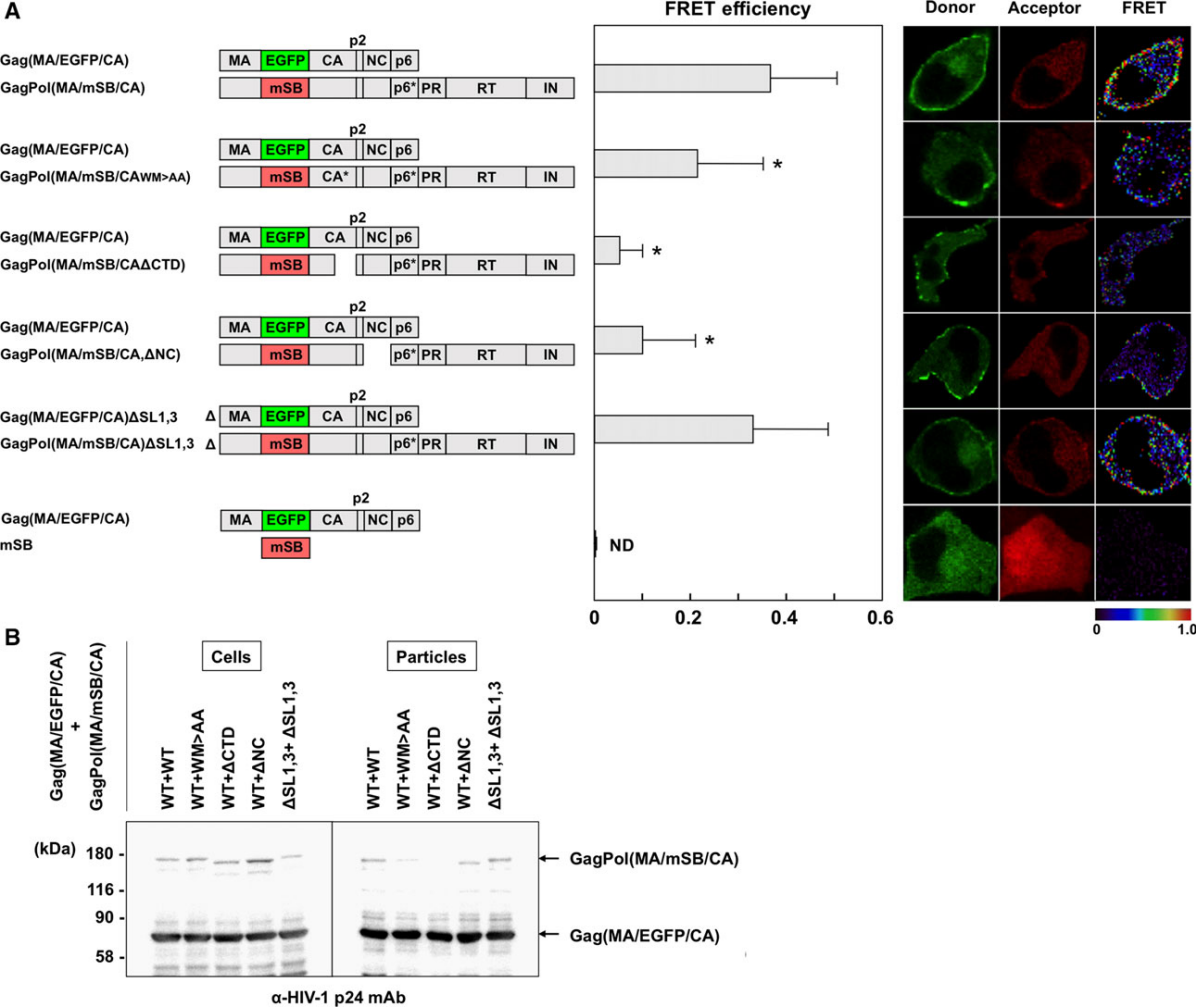
FRET assays for interactions of Gag and GagPol with mutations and virus particle production. (A) FRET efficiencies of Gag(MA/EGFP/CA) and GagPol(MA/mSB/CA) containing mutations. HeLa cells were cotransfected with Gag(MA/EGFP/CA) plus GagPol(MA/mSB/CA) molecular clone containing CA mutations (W184A and M185A) or a deletion of the CA CTD (ΔCA CTD), NC (ΔNC), or SL1 and 3 (ΔSL1,3) at a DNA ratio of 1 : 1. A combination of Gag(MA/EGFP/CA) and soluble mSB was used as the negative control. WM > AA, W184A and M185A. The FRET efficiencies of the donor Gag–acceptor GagPol pairs were statistically greater than that of the negative control (*P *<* *0.01). *Statistically significant (*P *<* *0.01) compared with the wild‐type Gag(MA/EGFP/CA)–GagPol(MA/mSB/CA) pair. Representative FRET images are shown. (B) Virus particle production. HeLa cells were cotransfected with Gag(MA/EGFP/CA) and GagPol(MA/mSB/CA) molecular clones at a DNA ratio of 10 : 1. The particle fractions purified from the cell culture supernatants were analyzed by western blotting using a monoclonal antibody specific for HIV‐1 p24CA.

Previous studies have suggested that the CA CTD, especially the region spanning the MHR and the adjacent CA C‐terminal sequences within the GagPol context, is responsible for the incorporation of GagPol into virus particles 12, 13. To confirm this, we made GagPol(MA/mSB/CA) constructs with the dimer interface mutations W184A and M185A, or the deletion of the CA CTD or NC, and examined coexpression with Gag(MA/EGFP/CA) (Fig. 4A). Their FRET efficiencies were reduced to varying degrees, that is, *E*_A_ = 0.21 for GagPol(MA/mSB/CA W184A,M185A), 0.06 for GagPol(MA/mSB/ΔCA CTD), and 0.11 for GagPol(MA/mSB/CA,ΔNC). These data indicated that deletion of the CA CTD had the most deleterious effect on Gag–GagPol interactions in our FRET system. We investigated the incorporation of these GagPol mutants in HIV particles (Fig. 4B). Western blotting indicated that the deletion of the CA CTD severely impaired the incorporation of GagPol(MA/mSB/CA) into particles but, in contrast, the deletion of NC and the mutations W184A and M185A showed almost no effects or slightly negative effects on the incorporation of GagPol(MA/mSB/CA).

Nucleocapsid contributes to Gag assembly by accumulating individual Gag molecules on HIV genomic RNA 11. To investigate if Gag–GagPol interactions involve RNA binding, the RNA dimerization initiation and packaging signals SL1 and 3 were deleted from both Gag(MA/EGFP/CA) and GagPol(MA/mSB/CA) constructs. The FRET efficiency of the ΔSL1,3 constructs was comparable to that produced by the wild‐type constructs Gag(MA/EGFP/CA) plus GagPol(MA/mSB/CA) (*E*_A_ = 0.32 versus 0.37; Fig. 4A). Western blotting showed that the coexpression of Gag(MA/EGFP/CA)ΔSL1,3 and GagPol(MA/mSB/CA)ΔSL1,3 produced nearly equivalent levels of virus particles as compared with the coexpression of Gag(MA/EGFP/CA) plus GagPol(MA/mSB/CA) (Fig. 4B). These data suggested that HIV genomic RNA had little or no impact on Gag–GagPol interactions and the subsequent incorporation of GagPol into virus particles. A role for nonspecific interactions of RNA with NC cannot be ruled out.

### FRET analysis of GagPol homodimerization

**Figure 5:**
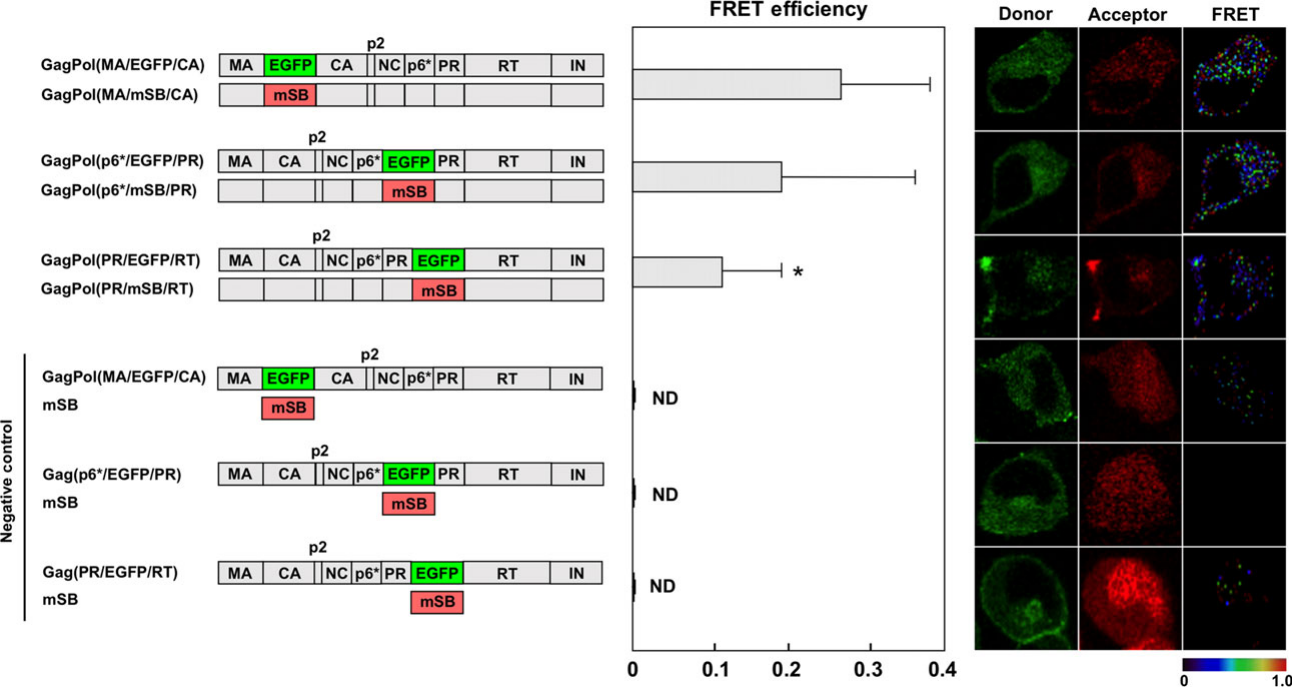
FRET assays for GagPol homodimerization with interdomain constructs. HeLa cells were cotransfected with GagPol(MA/F/CA), GagPol(p6*/F/PR), or GagPol(PR/F/RT) molecular clone pair supplemented with authentic Gag‐expressing construct (donor GagPol: acceptor GagPol: Gag = 1 : 1 : 2). Combinations of GagPol(MA/EGFP/CA), GagPol(p6*/EGFP/PR), or GagPol(PR/F/RT) molecular clone and a plasmid expressing mSB were used as negative controls. The FRET efficiencies were statistically greater than those of the corresponding negative controls (*P *<* *0.01). *Statistically significant (*P *<* *0.01) as compared with the GagPol(MA/F/CA) pair. Representative FRET images are shown.

The Gag region is capable of multimerization and the Pol region also exhibits dimerization activity in the context of GagPol. It is widely accepted that the homodimerization of GagPol is required for the autocleavage of GagPol precursors. Fluorescent proteins were inserted at the MA–CA, p6*–PR, and PR–RT junctions in the pNL4‐3 construct with the frameshift mutation and inactive PR (Fig. 5). These EGFP‐ and mSB‐expressing GagPol pairs were coexpressed with authentic Gag, as GagPol dimerization occurs only after plasma membrane targeting with Gag 25, 26. The FRET efficiency by GagPol(MA/F/CA) was ~ 0.26. The FRET efficiency by GagPol(p6*/F/PR) was 0.19, whereas that by GagPol(PR/F/RT) was 0.11. These efficiencies were considerably low but were still significant in comparison with corresponding negative controls (*E*_A_ = 0, 0, and 0.004, respectively).

## Discussion

### Gag and Pol interdomains tolerant to fluorescent protein insertion for FRET assays

Recent cryoelectron microscopy and tomography studies have revealed the radial density profiles of immature HIV particles with three peaks of high density corresponding to the membrane‐MA, CA, and NC–RNA layers, with spacing of 4 and 3 nm 33, 34, 35, 36. These data suggest that the MA–CA and CA–NC interdomain regions are not densely packed. In fact, the pNL4‐3 derivative containing fluorescent proteins at the MA–CA junction was replication‐competent and analyzed by FRET assays 20, 29. However, the availability of the Gag terminal and internal fluorescent constructs for FRET assays has not been evaluated in parallel.

We placed EGFP and mSB at the MA–CA junction and performed FRET assays. In Gag–Gag interaction assays, the FRET efficiency of Gag(MA/F/CA) was 0.58 (Fig. 1). When the CA dimer interface was disrupted by the mutations W184A and M185A, the FRET efficiency of the mutant Gag(MA/F/CA) was reduced, consistent with a previous study with Gag(p6/CFP and YFP) 21. The authors suggested the formation of a half‐interface with a comparable FRET efficiency when the mutations were present only in the acceptor Gag, but did not test the reverse combination. We found a reduction only when the mutations were present in the donor Gag, but no reduction when they were present only in the acceptor Gag (Fig. 2). It is possible that the relative orientation of donor and acceptor dipole moments was not parallel, due to subtle structural alterations in the donor, especially when the mutations were involved. Using GagPol(MA/F/CA), we also investigated Gag–GagPol and GagPol–GagPol interactions and found that their FRET efficiencies (*E*_A_ = 0.37 and 0.26, respectively) were significantly different from those of their negative controls (Figs 3 and 5). These data suggest that fluorescent proteins inserted at the MA–CA junction were available for FRET analyses of Gag–Gag as well as Gag–GagPol and GagPol–GagPol interactions.

For comparison, we used Gag(p6/F) constructs with fluorescent proteins at the Gag C terminus and GagPol(p6*/F/PR) with fluorescent proteins at the p6*–PR junction. As structural studies have indicated that both p6 and p6* are highly flexible 37, 38, the addition/insertion of fluorescent proteins was not expected to cause steric hindrance. The Gag(p6/F) and GagPol(p6*/F/PR) constructs exhibited FRET efficiencies for Gag–Gag (*E*_A_ = 0.58) and Gag–GagPol (*E*_A_ = 0.36) that were equivalent or comparable to those for Gag(MA/F/CA) and GagPol(MA/F/CA) constructs (Figs 1 and 3). However, when the GagPol(p6*/F/PR) constructs were used for GagPol–GagPol interactions, the FRET efficiency was apparently lower than that obtained for the GagPol(MA/F/CA) constructs (Fig. 5). Some reports have shown that the p6* domain suppresses PR activation and dimerization 39, 40, suggesting that the insertion just upstream of PR is critical for GagPol dimerization.

Fluorescent proteins were also placed at the p2–NC junction in this study. When the Gag(p2/F/NC) and GagPol(p2/F/NC) constructs were used, the FRET efficiencies for Gag–Gag and Gag–GagPol interactions were significantly lower (*E*_A_ = 0.06 and 0.17, respectively) than those for the constructs with fluorescent proteins at the MA–CA or the C‐terminal p6 (0.58 and 0.36‐0.37, respectively; Figs 1 and 3). Because several Gag–Gag interaction sites are present around the p2–NC junction (e.g., CA CTD, and NC) 5, 11, 30, 31, 32, the insertion of the p2–NC junction was most likely to severely impair Gag–Gag and Gag–GagPol interactions. Together, our study indicated that the insertion at the MA–CA junction had the least deleterious effect on Gag–Gag and Gag–GagPol interactions.

### Gag–GagPol interaction

Deletion of the CA CTD in GagPol severely reduced the FRET for Gag–GagPol interactions and GagPol incorporation into HIV particles (Fig. 4). However, the mutations W184A and M185A in CA helix 9 showed a mild or very faint reduction in the FRET efficiency and GagPol incorporation into particles. As one study has reported that the deletion of CA helix 9 in GagPol abolishes its particle incorporation 14, it is possible that the entire CA helix 9, not specific amino acids alone, was involved in Gag–GagPol interactions. Alternatively, the formation of a half‐interface of CA helix 9 may have rescued the particle incorporation of GagPol. Similarly, the deletion of NC in GagPol significantly reduced FRET for Gag–GagPol interactions, but did not impair GagPol incorporation into particles. Based on these findings, it is possible that Gag–GagPol interactions occurred via Gag regions other than the insertion site of the fluorescent protein, so that GagPol was incorporated into particles.

### Dimerization of GagPol precursors

We and others have previously reported FRET systems for GagPol dimerization with Gag/Pol‐F constructs (F, a combination of CFP and YFP, or EGFP and mSB), in which fluorescent proteins were placed at the PR–RT junction 22, 23, 26. In the present study, we tested three interdomains in GagPol (the MA–CA, p6*–PR, and PR–RT junctions) to insert fluorescent proteins. Their FRET efficiencies were generally low as compared with the FRET efficiencies for Gag dimerization, but were as follows: MA–CA (*E*_A_ = 0.26) > p6*–PR (*E*_A_ = 0.19) > PR–RT (*E*_A_ = 0.11). Although the overall ternary structure of the GagPol precursor has not been solved by crystallography, many dimerization or oligomerization domains within GagPol have been identified, including trimerization via MA, hexamerization via the CA NTD, dimerization via the CA CTD, PR and RT and tetramerization via IN 4, 5, 7, 16, 17, 18. It is still not clear which domains play major roles in GagPol dimerization, but it is possible that steric hindrance caused by fluorescent protein insertion may have weakened dimerization of the adjacent N‐ and CTDs in the context of GagPol precursor. Based on this hypothesis, our data may suggest that Pol interdomain insertions have more deleterious effects on GagPol dimerization than Gag interdomain insertions.

## Author contributions

YM conceived and supervised the study; ST and YM performed experiments; ST and FM analyzed the data; ST, YM, and FM wrote the manuscript.

## References

[1] Gheysen D , Jacobs E , de Foresta F , Thiriart C , Francotte M , Thines D and de Wilde M (1989) Assembly and release of HIV‐1 precursor Pr55gag virus‐like particles from recombinant baculovirus‐infected insect cells. Cell 59, 103–112. 2676191 10.1016/0092-8674(89)90873-8

[2] Haraguchi H , Noda T , Kawaoka Y and Morikawa Y (2012) A large extension to HIV‐1 Gag, like Pol, has negative impacts on virion assembly. PLoS ONE 7, e47828. 23110110 10.1371/journal.pone.0047828 PMC3479142

[3] Smith AJ , Srinivasakumar N , Hammarskjold ML and Rekosh D (1993) Requirements for incorporation of Pr160^*gag‐pol*^ from human immunodeficiency virus type 1 into virus‐like particles. J Virol 67, 2266–2275. 8445731 10.1128/jvi.67.4.2266-2275.1993 PMC240363

[4] Tang C , Loeliger E , Luncsford P , Kinde I , Beckett D and Summers MF (2004) Entropic switch regulates myristate exposure in the HIV‐1 matrix protein. Proc Natl Acad Sci USA 101, 517–522. 14699046 10.1073/pnas.0305665101 PMC327179

[5] Gamble TR , Yoo S , Vajdos FF , von Schwedler UK , Worthylake DK , Wang H , McCutcheon JP , Sundquist WI and Hill CP (1997) Structure of the carboxyl‐terminal dimerization domain of the HIV‐1 capsid protein. Science 278, 849–853. 9346481 10.1126/science.278.5339.849

[6] von Schwedler UK , Stray KM , Garrus JE and Sundquist WI (2003) Functional surfaces of the human immunodeficiency virus type 1 capsid protein. J Virol 77, 5439–5450. 12692245 10.1128/JVI.77.9.5439-5450.2003 PMC153941

[7] Li S , Hill PC , Sundquist IW and Finch TJ (2000) Image reconstructions of helical assemblies of the HIV‐1 CA protein. Nature 407, 409–413. 11014200 10.1038/35030177

[8] Pornillos O , Ganser‐Pornillos BK , Kelly BN , Hua Y , Whitby FG , Stout CD , Sundquist WI and Hill CP (2009) X‐ray structures of the hexameric building block of the HIV capsid. Cell 137, 1282–1292. 19523676 10.1016/j.cell.2009.04.063 PMC2840706

[9] Zhao G , Perilla JR , Yufenyuy EL , Meng X , Chen B , Ning J , Ahn J , Gronenborn AM , Schulten K , Aiken C *et al* (2013) Mature HIV‐1 capsid structure by cryo‐electron microscopy and all‐atom molecular dynamics. Nature 497, 643–646. 23719463 10.1038/nature12162 PMC3729984

[10] De Guzman RN , Wu ZR , Stalling CC , Pappalardo L , Borer PN and Summers MF (1998) Structure of the HIV‐1 nucleocapsid protein bound to the SL3‐RNA recognition element. Science 279, 384–388. 9430589 10.1126/science.279.5349.384

[11] Alfadhli A , Dhenub TC , Still A and Barklis E (2005) Analysis of human immunodeficiency virus type 1 Gag dimerization‐induced assembly. J Virol 79, 14498–14506. 16282449 10.1128/JVI.79.23.14498-14506.2005 PMC1287545

[12] Huang M and Martin MA (1997) Incorporation of Pr160^*gag‐pol*^ into virus particles requires the presence of both the major homology region and adjacent C‐terminal capsid sequences within the Gag‐Pol polyprotein. J Virol 71, 4472–4478. 9151838 10.1128/jvi.71.6.4472-4478.1997 PMC191666

[13] Chiu HC , Yao SY and Wang CT (2002) Coding sequences upstream of the human immunodeficiency virus type 1 reverse transcriptase domain in Gag‐Pol are not essential for incorporation of the Pr160^*gag‐pol*^ into virus particles. J Virol 76, 3221–3231. 11884546 10.1128/JVI.76.7.3221-3231.2002 PMC136043

[14] Chien AI , Liao WH , Yang DM and Wang CT (2006) A domain directly C‐terminal to the major homology region of human immunodeficiency type 1 capsid protein plays a crucial role in directing both virus assembly and incorporation of Gag‐Pol. Virology 348, 84–95. 16442581 10.1016/j.virol.2005.12.009

[15] Louis JM , Clore GM and Gronenborn AM (1999) Autoprocessing of HIV‐1 protease is tightly coupled to protein folding. Nat Struct Biol 6, 868–875. 10467100 10.1038/12327

[16] Naiva MA , Fitzgrald PM , Mckeever BM , Leu CT , Heimbach JC , Sigal IS , Darke PL and Springer JP (1989) Three‐dimensional structure of aspartyl‐protease from human immunodeficiency virus HIV‐1. Nature 337, 615–619. 2645523 10.1038/337615a0

[17] Kohlstaedt LA , Wan J , Friedman JM , Rice PA and Steitz TA (1992) Crystal structure at 3.5 A resolution of HIV‐1 reverse transcriptase complexed with an inhibitor. Science 256, 1783–1790. 1377403 10.1126/science.1377403

[18] Hare S , Gupta SS , Valkov E , Engelman A and Cherepanov P (2010) Retroviral intasome assembly and inhibition of DNA strand transfer. Nature 464, 232–236. 20118915 10.1038/nature08784 PMC2837123

[19] Derdowski A , Ding L and Spearman P (2004) A novel fluorescence resonance energy transfer assay demonstrates that the human immunodeficiency virus type 1 Pr55Gag I domain mediates Gag‐Gag interactions. J Virol 78, 1230–1242. 14722278 10.1128/JVI.78.3.1230-1242.2004 PMC321371

[20] Hübner W , Chen P , Del Portillo A , Liu Y , Gordon RE and Chen BK (2007) Sequence of human immunodeficiency virus type 1 (HIV‐1) Gag localization and oligomerization monitored with live confocal imaging of a replication‐competent, fluorescently tagged HIV‐1. J Virol 81, 12596–12607. 17728233 10.1128/JVI.01088-07 PMC2168995

[21] Hogue IB , Hoppe A and Ono A (2009) Quantitative fluorescence resonance energy transfer microscopy analysis of the human immunodeficiency virus type 1 Gag‐Gag interaction: relative contributions of the CA and NC domains and membrane binding. J Virol 83, 7322–7336. 19403686 10.1128/JVI.02545-08 PMC2704781

[22] Koh Y , Matsumi S , Das D , Amano M , Davis DA , Li J , Leschenko S , Baldridge A , Shioda T , Yarchoan R *et al* (2007) Potent inhibition of HIV‐1 replication by novel non‐peptidyl small molecule inhibitors of protease dimerization. J Biol Chem 282, 28709–28720. 17635930 10.1074/jbc.M703938200

[23] Koh Y , Aoki M , Danish ML , Aoki‐Ogata H , Amano M , Das D , Shafer RW , Ghosh AK and Mitsuya H (2011) Loss of protease dimerization inhibition activity of darunavir is associated with the acquisition of resistance to darunavir by HIV‐1. J Virol 85, 10079–10089. 21813613 10.1128/JVI.05121-11 PMC3196396

[24] Venezia CF , Meany BJ , Braz VA and Barkley MD (2009) Kinetics of association and dissociation of HIV‐1 reverse transcriptase subunits. Biochemistry 48, 9084–9093. 19715314 10.1021/bi9010495 PMC2770954

[25] Haraguchi H , Sudo S , Noda T , Momose F , Kawaoka Y and Morikawa Y (2010) Intracellular localization of human immunodeficiency virus type 1 Gag and GagPol products and virus particle release: relationship with the Gag‐to‐GagPol ratio. Microbiol Immunol 54, 734–746. 21091985 10.1111/j.1348-0421.2010.00276.x

[26] Sudo S , Haraguchi H , Hirai Y , Gatanaga H , Sakuragi J , Momose F and Morikawa Y (2013) Efavirenz enhances HIV‐1 Gag processing on the plasma membrane through GagPol dimerization. J Virol 87, 3348–3360. 23302874 10.1128/JVI.02306-12 PMC3592135

[27] Clever JL and Parslow TG (1997) Mutant human immunodeficiency virus type 1 genomes with defects in RNA dimerization or encapsidation. J Virol 71, 3407–3414. 9094610 10.1128/jvi.71.5.3407-3414.1997 PMC191485

[28] van Rheenen J , Langeslag M and Jalink K (2004) Correcting confocal acquisition to optimize imaging of fluorescence resonance energy transfer by sensitized emission. Biophys J 86, 2517–2529. 15041688 10.1016/S0006-3495(04)74307-6 PMC1304099

[29] Müller M , Daecke J , Oliver T , Fackler OT , Dittmar MT , Zentgraf H and Kräusslich HG (2004) Construction and characterization of a fluorescently labeled infectious human immunodeficiency virus type 1 derivative. J Virol 78, 10803–10813. 15367647 10.1128/JVI.78.19.10803-10813.2004 PMC516407

[30] Accola MA , Strack B and Göttlinger HG (2000) Efficient particle production by minimal Gag constructs which retain the carboxy‐terminal domain of human immunodeficiency virus type 1 capsid‐p2 and a late assembly domain. J Virol 74, 5395–5402. 10823843 10.1128/jvi.74.12.5395-5402.2000 PMC112023

[31] Datta SA , Temeselew LG , Crist RM , Soheilian F , Kamata A , Mirro J , Harvin D , Nagashima K , Cachau RE and Rein A (2011) On the role of the SP1 domain in HIV‐1 particle assembly: a molecular switch? J Virol 85, 4111–4121. 21325421 10.1128/JVI.00006-11 PMC3126284

[32] Schur FK , Obr M , Hagen WJ , Wan W , Jakobi AJ , Kirkpatrick JM , Sachse C , Kräusslich HG and Briggs JA (2016) An atomic model of HIV‐1 capsid‐SP1 reveals structures regulating assembly and maturation. Science 353, 506–508. 27417497 10.1126/science.aaf9620

[33] Wright ER , Schoolerm JB , Ding HJ , Kieffer C , Fillmore C , Sundquist WI and Jensen GJ (2007) Electron cryotomography of immature HIV‐1 virions reveals the structure of the CA and SP1 Gag shells. EMBO J 26, 2218–2226. 17396149 10.1038/sj.emboj.7601664 PMC1852790

[34] Briggs JA , Riches JD , Glass B , Bartonova V , Zanetti G and Kräusslich HG (2009) Structure and assembly of immature HIV. Proc Natl Acad Sci USA 106, 11090–11095. 19549863 10.1073/pnas.0903535106 PMC2700151

[35] Bharat TA , Davey NE , Ulbrich P , Riches JD , de Marco A , Rumlova M , Sachse C , Ruml T and Briggs JA (2012) Structure of the immature retroviral capsid at 8 Å resolution by cryo‐electron microscopy. Nature 487, 385–389. 22722831 10.1038/nature11169

[36] Schur FK , Hagen WJ , Rumlova M , Ruml T , Müller B , Kräusslich HG and Briggs JA (2015) Structure of the immature HIV‐1 capsid in intact virus particles at 8.8 Å resolution. Nature 517, 505–508. 25363765 10.1038/nature13838

[37] Fossen T , Wray V , Bruns K , Rachmat J , Henklein P , Tessmer U , Maczurek A , Klinger P and Schubert U (2005) Solution structure of the human immunodeficiency virus type 1 p6 protein. J Biol Chem 280, 42515–42527. 16234236 10.1074/jbc.M507375200

[38] Beissinger M , Paulus C , Bayer P , Wolf H , Rosch P and Wagner R (1996) Sequence‐specific resonance assignments of the 1H‐NMR spectra and structural characterization in solution of the HIV‐1 transframe protein p6*. Eur J Biochem 237, 383–392. 8647076 10.1111/j.1432-1033.1996.0383k.x

[39] Partin K , Zybarth G , Ehrlich L , DeCrombrugghe M , Wimme E and Carter C (1991) Deletion of sequences upstream of the proteinase improves the proteolytic processing of human immunodeficiency virus type 1. Proc Natl Acad Sci USA 88, 4776–4780. 1647017 10.1073/pnas.88.11.4776 PMC51749

[40] Zybarth G and Carter C (1995) Domains upstream of the protease (PR) in human immunodeficiency virus type 1 Gag‐Pol influence PR autoprocessing. J Virol 69, 3878–3884. 7745738 10.1128/jvi.69.6.3878-3884.1995 PMC189109

